# Prevalence of multidrug resistance, extensive drug resistance and pandrug resistance among multiple Gram-negative isolates: experience in a tertiary-care hospital ICU in North India

**DOI:** 10.1186/cc12014

**Published:** 2013-03-19

**Authors:** S Dewan, T Sahoo, N Chandra, A Varma

**Affiliations:** 1 Fortis Escorts Heart Institute, New Delhi, India

## Introduction

Antibiotic resistance is a major worldwide problem in the ICU [1]. The situation in developing countries like India is particularly serious. Since the presence of drug-resistant bacteria in the environment is a threat for public health, up-to-date information on local pathogens and the drug sensitivity pattern is very crucial to treat patients. This study was carried out to evaluate the prevalence of multidrug resistance (MDR), extensive drug resistance (XDR) and pandrug resistance (PDR) among multiple Gram-negative isolates in a medical-surgical ICU in a tertiary care hospital in North India.

## Methods

We conducted a prospective observational study. All data were analysed using descriptive statistics. All Gram-negative culture isolates over a period of 13 months (October 2011 to October 2012) were included in this study. Isolation and identification were performed using the bact alert system and VITEK2, respectively. Sensitivities were determined by Kirby Bauer disc diffusion and broth dilution using VITEK2-AST cards and interpreted according to Clinical and Laboratory Standards Institute criteria. For the purpose of this study, we used MDR to denote isolates resistant to representatives three or more classes of antimicrobial agents, XDR as those resistant to all but one or two classes and PDR as those resistant to all classes of antimicrobial agents available [2,3].

## Results

**Figure 1:**
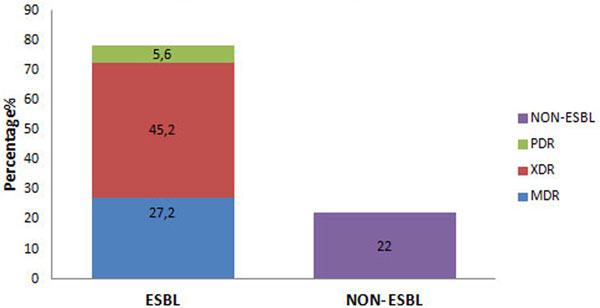
**Prevalence of drug-resistant Gram-negative isolates**.

**Figure 2:**
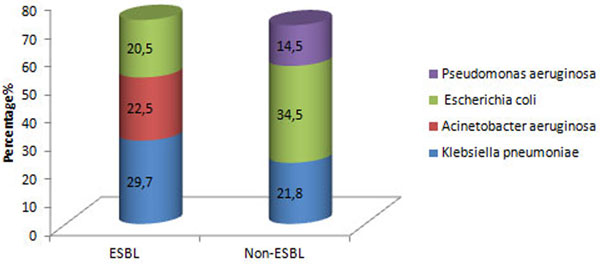
**Prevalence of Gram-negative isolates**.

Out of a total 2,796 culture specimens sent over 13 months, 250 isolates were Gram-negative (8.9%). Among these 250 (*n*) Gram-negative isolates, 195 (78%) were extended-spectrum ß-lactamase (ESBL) producers and the remaining 55 (22%) were non-ESBL producers. Among the ESBL producers, PDR, XDR and MDR isolates were 14 (5.6%), 113 (45.2%) and 68 (27.2%), respectively (Figure 1). Among the XDR-positive organisms, seven (6.1%) organisms were New Delhi metallo-ß-lactamase-1 (NDM-1) producers and five (4.4%) organisms were NDM-2 producers. Among ESBL-positive isolates, the most predominant isolate was *Klebsiella pneumoniae *(29.7%) followed by *Acinetobacter aeruginosa *(22.5%) and *Escherichia coli *(20.5%) (Figure 2). Among non-ESBL-positive isolates, the most predominant isolate was *Escherichia coli *(34.5%) followed by *Klebsiella pneumoniae *(21.8%) and *Pseudomonas aeruginosa *(14.5%) (Figure 2).

## Conclusion

ESBL producers were the most frequently isolated Gram-negative bacterial isolates in this tertiary-care hospital in north India.

Among ESBL producers, XDR organisms were most frequent, followed by MDR and PDR organisms. Few of the XDR isolates were NDM producers, which have propensity to spread to other bacteria. In view of significant prevalence of multidrug resistance amongst Gram-negative organisms in the ICU, regular surveillance of antibiotic susceptibility patterns plays a crucial role for setting orders to guide the clinician in choosing empirical or directed therapy of infected patients.
